# Assessment of Landslide Activity and Hazard Recorded in Tree Rings—Examples from the High-Risk Vernazza Catchment (Liguria, Italy)

**DOI:** 10.3390/s25237304

**Published:** 2025-12-01

**Authors:** Ireneusz Malik, Michael Maerker, Małgorzata Wistuba, Elżbieta Gorczyca, Patrizio Torrese, Priscilla Niyokwiringirwa, Yang Yu, Beata Woskowicz-Ślęzak, Anna Bieniasz

**Affiliations:** 1 Polish-Chinese Centre for Environmental Research, Institute of Earth Sciences, University of Silesia in Katowice, 60, Będzińska, 41-200 Sosnowiec, Polandbeata.woskowicz-slezak@us.edu.pl (B.W.-Ś.);; 2 Working Group on Soil Erosion and Feedbacks, Leibniz Centre for Agricultural Landscape Research (ZALF), Eberswalder Straße 84, 15374 Muencheberg, Germany; 3 Department of Earth and Environmental Sciences, University of Pavia, Via Ferrata 1, 27100 Pavia, Italy; patrizio.torrese@unipv.it; 4 Institute of Geography and Spatial Management, Jagiellonian University in Kraków, Gronostajowa 7, 30-387 Kraków, Poland; 5 Department of Earth, Environment and Life Science (DISTAV), University of Genova, Corso Europa 26, 27100 Genoa, Italy; 6 State Key Laboratory of Ecological Safety and Sustainable Development in Arid Lands, Xinjiang Institute of Ecology and Geography, Chinese Academy of Sciences, Urumqi 830011, China

**Keywords:** natural hazards, landslide activity, deep-seated landslides, tree rings, eccentricity index

## Abstract

**Highlights:**

**What are the main findings?**

- For two landslides in one catchment, dominant landslide events occurred in different years, but the general trend of landslide activity is the same.
- There is no high risk of sudden triggering of the studied landslides.

**What are the implications of the main findings?**

- Information on landslide activity and hazard should be generalised with caution, even within a single catchment.
- Dendrochronology allow to estimate landslide activity and hazard.

**Abstract:**

In the Vernazza catchment located in Italian Liguria, we studied the activity and hazard of two deep-seated landslides using tree rings. Knowledge of the deep-seated landslide movements in terms of their temporal activation is still scarce in the Vernazza catchment, as well as in similar catchments. To determine the hazard of the studied landslides, we compared the landslide activity for landslides under study with landslides from Poland that activated in 2010. Within the two studied Italian landslides, increased landslide activity occurred at similar times, but the dominant episodes occurred in different years. It means that the information on landslide activity and hazard should be generalised with caution, even within a single catchment. The studied deep-seated landslide activity was significantly higher after the landslide catastrophe in 2011, when many shallow landslides were activated in the studied catchment. The increase in landslide activity in 2011 was accompanied by a substantial transformation of the catchment’s relief. These changes may have resulted in a loss of stability of deep-seated landslides. The landslide activity recorded by trees did not show an increase, unlike what was reported for the Polish landslides. This indicates that there is no high risk of sudden triggering of the studied landslides.

## 1. Introduction

Landslides are widespread mass movements, particularly occurring in the Apennines along the Italian peninsula. Their occurrence often causes significant destruction and millions of Euros in losses [1,2,3]. Many times, shallow landslides occur during heavy rainfall, and related material moves into the lower, often built-up parts of the catchment. The transported material may damage buildings, cars, roads, and other infrastructure [4,5]. Sometimes, extreme precipitation events are perceived as floods, but most of the damage occurs due to the transport of regolith, bedrock material, fluvial deposit, and vegetation debris from the upper reaches of the catchments [2,6]. One such disaster occurred in Italy in May 2023 in Emilia-Romagna, where 400 catastrophic shallow landslides were documented [7,8,9]. A similar catastrophe, but in a smaller area, occurred in the Vernazza catchment on 25 October 2011. The disaster led to the death of three people and severe damage to infrastructure in the lower parts of the catchment [10,11,12]. The area is part of the Cinque Terre and was included in the UNESCO list in 1997. Hence, the Vernazza catchment is of particular interest, and therefore, studies are conducted to understand the causes of the 2011 disaster and protect the area against future landslides and flood catastrophes [13,14,15].

One major problem during the 2011 disaster was the occurrence of 473 shallow landslides/debris slides that delivered material downstream. The material was derived primarily from the artificially terraced slopes [12,16,17]. The terraces are built up by dry stonewalls that are, for large parts, no longer maintained. At the same time, there are deep landslides (we use the term “deep-seated landslides” in the paper to separate shallow landslides/debris slides which occurred in the 2011 disaster and deep-seated landslides with a slip plane depth of over 5 metres, scarps, blocks and hummocky topography) in some parts of the catchment that were not triggered by the 2011 disaster, probably because the precipitation event that generated the disaster was heavy and short-lived, and prevalently produced surface runoff [13,18,19].

The knowledge of these deep-seated landslide movements, particularly in terms of their temporal activation frequency, remains scarce in the Vernazza catchment and in other catchments with similar characteristics in the Apennines. We propose a dendrochronological method to analyse landslide activity with annual accuracy, deriving valuable and crucial information regarding the stable conditions of these deep-seated landslides [20,21,22,23]. Trees tilting under the influence of landslide movement develop eccentric growth, reaction wood, and reductions in growth [24,25,26]. These features of wood anatomy allow the dating of landslide activity in particular years [27,28]. Using the dendrochronological method described by Malik et al. [29], we also assessed the possibility of future activation of the two selected deep-seated landslides.

Thus, this study aims to investigate the activity of two deep-seated landslides in the Vernazza catchment following the catastrophic episode of October 2011 (a) and to assess the related hazard of the studied landslides (b). 

## 2. Materials and Methods

**Figure 1:**
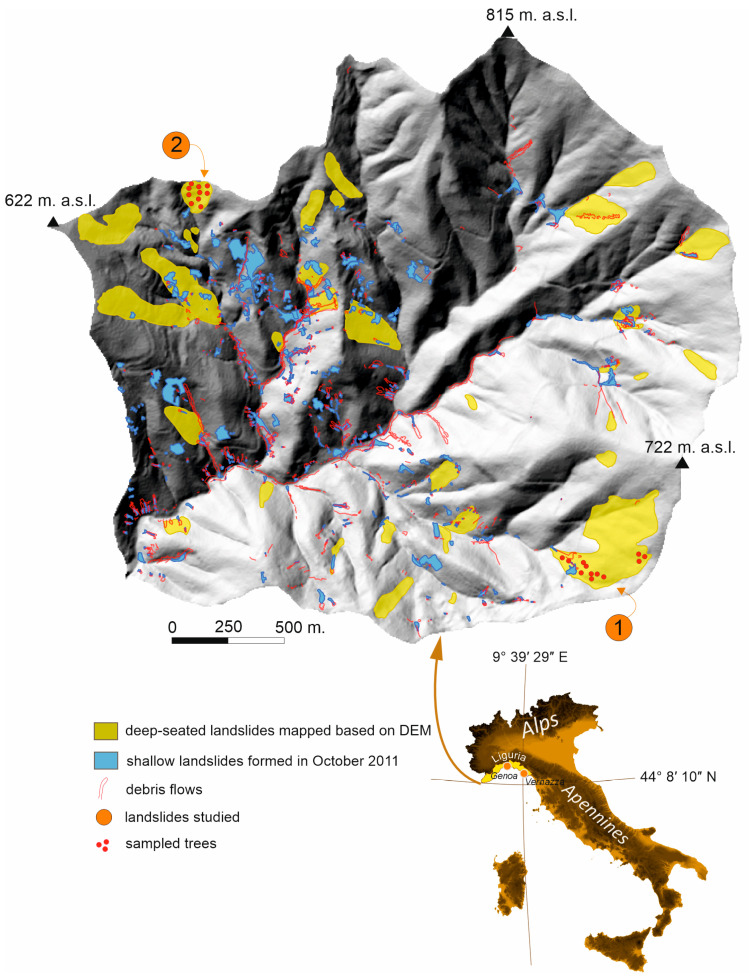
Location of the shallow landslides formed after the 2011, deep-seated landslides, and the two studied landslides in the Vernazza catchment.

The Vernazza catchment covers 5.7 km^2^ and is located in northern Italy on the Ligurian coast south east of Genoa (Figure 1). It is characterised by very steep slopes and short linear streams, mainly controlled by tectonics. The bedrock consists of a flysch composed of sandstone and claystone, known as the Macigno Formation, and a pelitic group that includes Canetolo Shales and Limestones [30,31]. The Eastern Liguria study area was affected by many heavy rainfall episodes or occasionally prolonged precipitation, which led to landslide formation approximately 2–4 times per decade [32,33,34,35,36,37].

**Figure 2:**
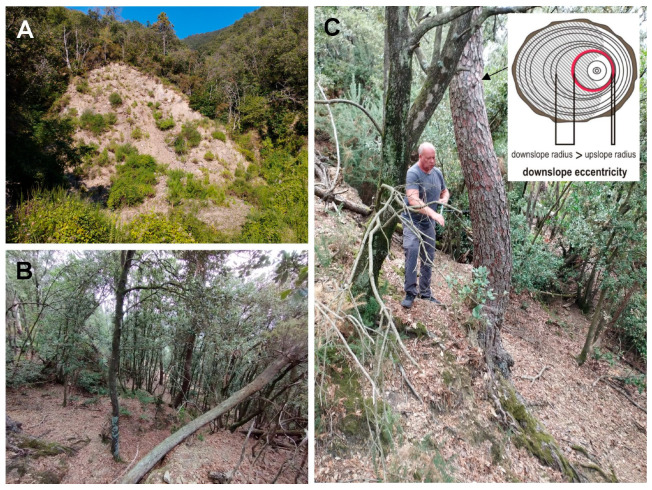
Landslide bodies and sampled trees growing on the landslides: (**A**)—shallow landslide formed in October 2011, (**B**)—surface of the landslide no. 2, and (**C**)—a sampled pine tilted by landsliding and cross-section of the tree stem showing tree ring eccentricity.

Most of the Vernaza catchment area is terraced for vineyards and olive groves, which were abandoned in the 20th century; only a few of these are still cultivated [38]. Terraces became unstable over the years due to a lack of maintenance, which caused them to be vulnerable to erosion [13]. Mainly shallow landslides and debris flows were formed during the 2011 catastrophe, and material from the destruction of the terraces was transported downstream [39] (Figure 1 and Figure 2). There are also deep-seated landslides in some parts of the catchment that were not triggered during the 2011 event but are visible on the DEM and in the field (Figure 1 and Figure 2). We identified 34 deep-seated landslides with a total area of 0.49 km^2^ (Figure 1). Steep scarps characterise these deep-seated landslides, numerous blocks, and hummocky topography (Figure 2B). The size of these landslides shows that a large amount of material is available to be transported down the catchment during subsequent rainfall episodes. The deep landslides are mainly overgrown with trees, including pines (*Pinus pinaster*).

In this study, the ring records of pine trees (Pinus pinaster) were analysed to determine the activity and hazard of two deep-seated landslides.

On landslide 1, we took 28 samples from 14 trees, and on landslide 2, we took 30 samples from 15 trees (Figure 1 and Figure 2C). We took two samples from each tree, one from the downslope side and one from the upslope side. We excluded from sampling trees that were injured, had more than one trunk, or showed signs of growth related to phototropism. Twenty samples were also taken from 10 trees on the reference slope, where no landslide occurs, located 2–3 km from the landslide slope no. 1. All samples were collected using a Pressler borer in the direction of the slope gradient.

The collected cores were glued into wooden holders and sanded with sandpaper to reveal distinct boundaries between the tree rings. Then, using an LINTAB measuring system (RINNTECH https://www.rinntech.com (accessed on 22 September 2025)), we measured the ring widths in the collected cores. Subsequently, we compared the curves by cross-dating the individual cores and identified the missing rings. Then, we calculated the yearly variation in the eccentricity index to determine landslide activity based on the previous studies [20,24,27,40].

Dating landslides activity using the calculation of the yearly variation in the eccentricity index involves analysing changes in ring width under the influence of landslides. When ground movement occurs, tree trunks growing on a slope tilt, causing a change in the radial growth of the tree trunks. The tree tries to correct its tilt by producing uneven rings—wider on one side and narrower on the other side (Figure 2C). In conifers (studied Pinus pinaster), wider rings are formed on the side toward which the tree is leaning. This difference in growth width is referred to as growth eccentricity. By analysing the sequences of tree rings and comparing the tree ring widths in the individual years for the downslope and upslope parts of a tree, we can identify the years in which eccentric rings occur. A previously developed mathematical formula was used to determine landslide episodes, assuming that landslides are identified by the eccentricity index (relation of the tree stem eccentricity to ring width for every following year expressed in percentage) and yearly variation in eccentricity is expressed as the yearly variation in the eccentricity index [24]:

E_x_ = U_x_ − D_x_.

Next, we recalculated the eccentricity into the eccentricity index:

if E_x_ > 0, then I_x_ = (E_x_/D_x_)*100%;

if E_x_ = 0, then I_x_ = E_x_*100%;

if E_x_ < 0, then I_x_ = (E_x_/U_x_)*100%;

And finally, we calculated the yearly variation in the eccentricity index:

V_x_ = I_x_ − I_x−1_

where E—eccentricity [mm]; U—ring width on its upslope side [mm]; D—ring width on its downslope side [mm]; I—eccentricity index [%]; V—yearly variation in eccentricity index [percent points]; x—year.

**Figure 3:**
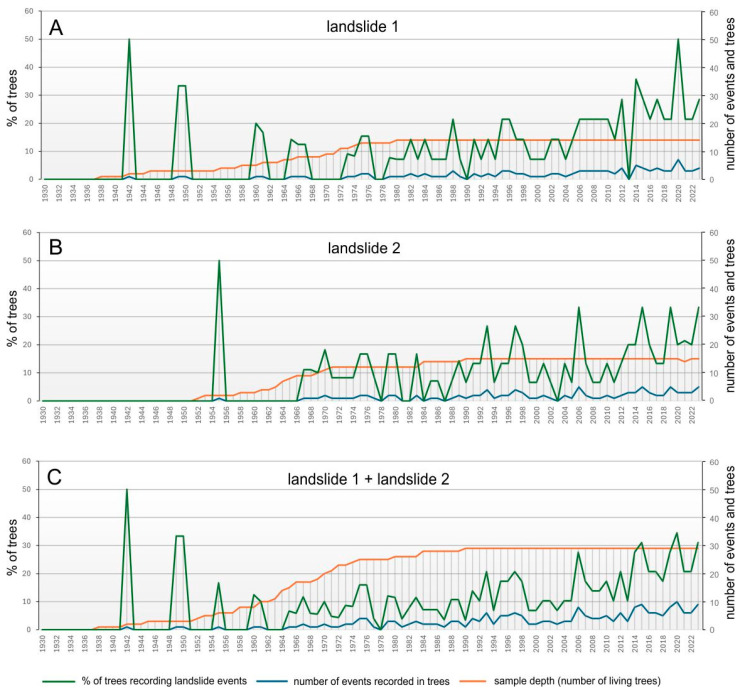
Landslide activities determined from tree rings: (**A**)—for landslide no. 1, (**B**)—for landslide no. 2, and (**C**)—for both studied landslides.

Studies that calculate the yearly variation in the eccentricity index typically use multiple trees to confirm the reliability of the results. The mathematical formula described above is applied to each tree individually. Other factors, such as wind or soil creep, can also cause eccentric tree growth. To separate the influence of these factors on tree growth, a stable (reference) slope is used, on which 10 samples are taken in the same manner as on the studied landslide slope. The reference slope is usually located as close as possible to the studied landslide slope. It should have characteristics as similar as possible to the studied landslide slope, e.g., geological structure, slope, etc. The yearly variation in eccentricity is calculated on the reference slope in the same way as on the landslide slope. It is assumed that all the yearly variations in the eccentricity index calculated for the landslide studied slope, which exceed the yearly variations in the eccentricity index on the reference slope, are the result of landsliding. The next step is to calculate the number of years with events that exceeded the yearly variation in the eccentricity index for each year. Then, absolute values are transformed into percentages of trees that recorded landslide events in each year. The final results are presented as the percentage of trees responding to landslides in each year (Figure 3).

Of course, the variability of climatic factors causing differentiation in ring widths throughout the entire circumference of the tree does not change the value of the yearly variation in the eccentricity index resulting from the tree’s inclination. The analysis uses the value of the difference in ring widths on the downslope and upslope sides of the tree, and this difference is not independent of the influence of climatic factors.

For the landslide hazard determination, we used a study conducted in Poland as a reference. The study was based on the tree ring pattern dynamics of two landslides (Siedloki landslide and Laski landslide) over 10 years preceding the landslide catastrophe in 2010. Laboratory analyses and measurements of tree rings for the sampled trees in the case of Polish and Italian landslides were also conducted in the same manner. For the calculation of the yearly variation in eccentricity index, we used the same mathematical formulas. Finally, we calculated the number of landslide events and the percentage of trees recording landslide events [24].

After the calculation, we found that trees growing on Polish landslides recorded an increase in landslide activity in the last few years before the disaster (before 2010). We assumed that if trees growing on landslides in the Vernazza catchment area also show a similar increase, a landslide disaster could occur in the future, as was the case in Poland. Even though climatic conditions, as well as vegetation and land use, are different, the lithology of both areas in Italy and Poland is very similar (mainly flysch-type lithology).

In the next stage of our work, we applied a mathematical procedure that enabled us to compare the yearly variation in the eccentricity index for the Siedloki and Łaski landslides with those located in the Vernazza catchment. For the Polish landslides, we determined the mean percentage of trees recording landsliding events for each of the 10 years preceding the 2010 landslide event. In addition, we calculated a reference (baseline) mean, representing the mean percentage of trees recording landsliding events from years earlier than the 10 years preceding the catastrophic landsliding, separately for the Siedloki and Laski sites. The 10-year interval (years 1–10 before the 2010 event) was selected because previous research indicates that trees can record a landslide hazard, as recorded by the yearly variation in the eccentricity index, considerably earlier than just 2–3 years before a landslide catastrophe [24]. The baseline mean was only derived for periods in which more than 85% of the sampled trees were alive. Next, we expressed the mean values for each of the 10 pre-landslide catastrophe years as a percentage relative to the baseline mean (again, calculated separately for the Siedloki and Laski landslides). This procedure enabled us to establish a characteristic pattern of dendrochronological response to the gradual acceleration of landslide movement for the 10 years preceding the landslide disaster (preparatory movements). Response pattern was subsequently applied to assess whether the Vernazza landslides could also have been triggered abruptly, in a manner comparable to the Siedloki and Laski events.

The same mathematical procedure was also applied to the landslides located in the Vernazza catchment. For these landslides, we determined the mean percentage of trees recording landsliding reactions for each of the last 10 years (2014–2023). The baseline value was established using the years preceding the 2014–2023 period. Finally, the results obtained for the Polish landslides were compared with those derived for the Vernazza landslides. Therefore, if the tree-ring patterns of the two Vernazza landslides resemble those observed in the Polish landslide, we might expect a catastrophic landslide activation. The method used to compare tree ring patterns between affected and non-affected landslides for catastrophic landslide hazards was described in detail by Malik et al. [29].

## 3. Results

The oldest of the sampled trees began growing in the 1920s and 1930s. Slightly older trees grew at landslide 1 and landslide 2; these trees started growing later, in the 1940s and 1950s. Therefore, the record of landslide events is 20 years longer at site 1. Statistically reliable results for the dataset studied were obtained from 1970 onwards. Since that year, landslide activity from the tree ring records of 70% of the sampled trees has been analysed. The most significant number of sampled trees, more than 30%, recorded landslide activity in 2015, 2020, and 2023 (Figure 3).

**Figure 4:**
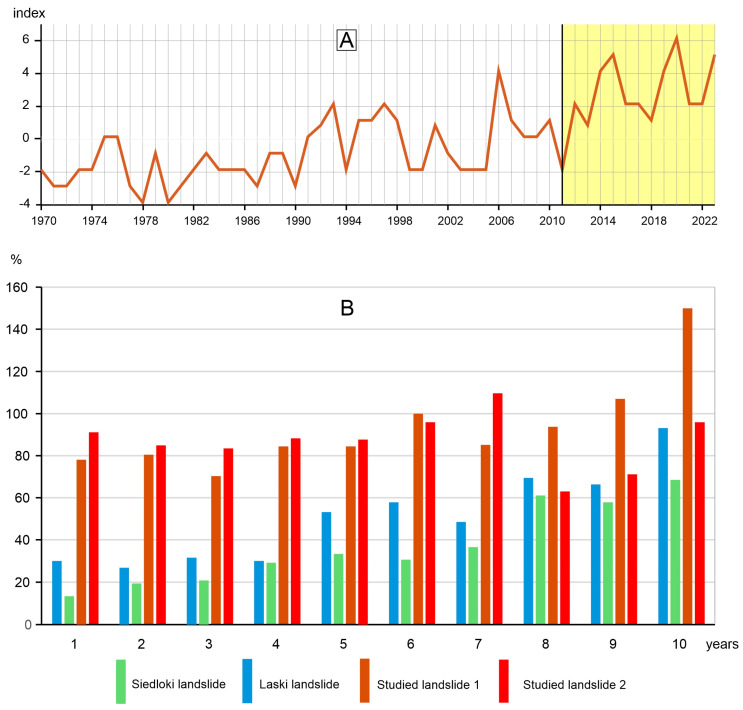
(**A**)—Graph showing an increase in landslide activity after the October 2011 catastrophic event. (**B**)—Landslide hazard compared for Siedloki and Laski (landslides from Poland) and landslides under study (no. 1 and no. 2). The research period covered 10 years before the landslide sudden slope failure in 2010 in the case of Siedloki and Laski landslides (numbers 1–10 on the horizontal axis); for landslides no. 1 and no. 2, the research period covered 10 years before 2024 (numbers 1–10 on the horizontal axis). Year after year percentage changes (vertical axis) of the mean values for each of the 10 pre-landslide catastrophe years (for Polish landslides) and 10 years before 2024 (for Italian landslides) in relation to the baseline mean (detailed explanation in the Section 2. Methods).

A significant landslide activity (with more than 20% of trees recorded events) was also found in 1993, 1997, and 2006 (Figure 3). At site 1, a very high activity was recorded in 2020, when a landslide affected half of the sampled trees (Figure 3). Based on the dendrochronological analysis, three periods of increased activity of the studied landslides can be distinguished. The first period spans 1964–1976, during which the number of living trees included in the landslide activity calculation was relatively small. The second period spans 1991–1998, and the third, the most extended period, covers 2006–2023 (Figure 3). Increased landslide activity can also be seen after the catastrophic episode that occurred in 2011 (Figure 4A).

Calculation of the landslide hazard showed that the possibility of catastrophic activation of landslide 2 is relatively low because we did not record systematic growth of tree reaction for a landslide in the last 10 years, as was the case of Polish landslides activated in 2010 (Figure 4B). Slightly different in activity is landslide number 2, where trees growing on the landslide recorded a significant increase in slow-scale landslide movement in 2023. However, the activity of the landslide was relatively stable in the past, and it has not increased year after year, unlike the case of the Polish landslides (Figure 4B).

## 4. Discussion

The studied landslides are located in the same catchment, but the activity of the two landslides varies despite similar geological and orographic conditions. For example, a very high landslide activity was recorded for landslide 1 in 2020, when 50% of the studied trees recorded landsliding. In turn, the activity recorded for landslide 2 involves only 20% of the responding trees in the same year. This means that, depending on local conditions, landslide movements are triggered in different years, even for nearby locations [41,42]. On the other hand, the same general changes in landslide activity over a more extended period are noticeable for both studied landslides; for both, we found an increase in landslide activity in the last decade of the 20th century. The study shows that even landslides located very close to each other can exhibit varying activity year after year. Hence, a comparison of the activity of landslides or groups of nearby landslides should be interpreted with caution. Special emphasis should be given to determine precipitation thresholds triggering landslides located in large areas [43,44].

Rainfall is the most critical factor causing landslides in Liguria [45]. Numerous shallow landslides were formed in Liguria during the catastrophic rainfall in October 2011. In turn, the trees sampled on the landslides recorded the highest activity in 2006, 2014, 2015, and 2020. In eastern Liguria, three heavy rainfall episodes were recorded in 2006, 2013, and 2014, resulting in numerous landslides [5,34,36,37,46]. These years correspond to the high activity of the studied landslides, as recorded dendrochronologically in 2006, 2014, and 2015. Activities of the studied landslides were also recorded in 2020, when the Liguria region was affected by first prolonged and then intense rainfall, with cumulative values in western Liguria exceeding 1000 mm in 45 days. The intensive precipitation event triggered approximately 1000 shallow landslides and debris flows, and also a few large, complex, deep-seated landslides occurred [35]. The high activity of the studied landslides, as determined by dendrochronology, appears to be attributed to these rainfall events. The rainfall events in 2006, 2014, 2015, and 2020 caused deep-seated landslide movements. Subsequently, these movements induced a tilting of the sampled trees, which in turn developed eccentric rings. This means that not only were shallow landslides triggered during high rainfall events, but there was also some activity of deep-seated landslides.

**Figure 5:**
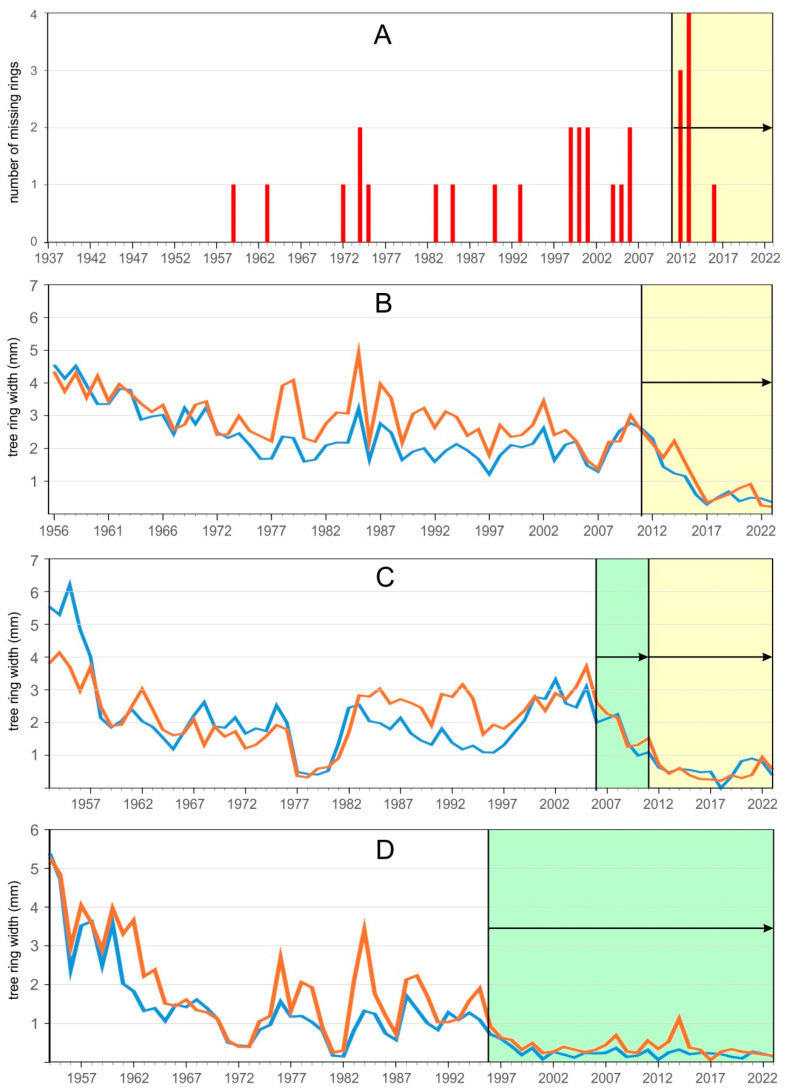
Missing rings and tree ring reductions recorded in the studied trees: (**A**)—missing rings (yellow colour showing missing rings formed after the 2011 event); (**B**)—upslope (blue curve) and downslope (red curve) measured from one of the studied trees (yellow colour showing ring reduction formed after the 2011); (**C**)—upslope (blue curve) and downslope (red curve) measured from one of the studied trees (yellow colour showing ring reduction formed after 2011 and green colour showing ring reduction formed from 2006); (**D**)—upslope (blue curve) and downslope (red curve) measured from one of the studied trees (green colour showing ring reduction formed from 1996).

What is astonishing is the lack of a landslide signal in the wood of the trees immediately after the Vernazza catchment disaster in 2011. No high landslide activity was recorded in 2012 and 2013. However, it was recorded in the studied trees from 2014 onwards. This phenomenon can be explained by the significant reduction in tree rings recorded after 2011 and the substantial number of missing rings in 2012–2013, which makes it impossible to calculate the tree eccentricity index for these years (Figure 5A–C). It appears that the catastrophic episode in 2011 damaged the root systems of the trees, leading to a reduction in their tree rings after 2011. A similar phenomenon was observed by Carrara and O’Neill [47] and Saez et al. [48].

Additionally, changes in the water table following the 2011 event may have contributed to reduced tree ring growth in the years following the severe 2011 event. Tree ring reduction occurred in the trees not only after the 2011 event, but also earlier (Figure 5C,D). The adverse impact of various environmental factors, such as insect outbreaks and air pollution, on the radial growth of trees and the consequent reduction in tree rings has been previously described [49,50]. In the case of the rings related to the studied landslides, the reduction can be attributed to the increase in temperature over recent decades, as recorded in various parts of northern Italy [51]. Weakened trees may be susceptible to insect outbreaks or other environmental factors that cause additional stress, resulting in even deeper reductions in tree rings and the appearance of missing rings [52,53].

Over the last decade, the trees growing on the studied landslides have shown a significant increase in landslide activity compared to the earlier period. It is evident in the form of significant values of landslide activity over the last 10 years, compared to the Polish landslides triggered during the 2010 catastrophe (Figure 4B). This may be due to the loss of stability of the studied landslide slopes during the 2011 catastrophic rainfall, but there is no clear evidence to support this.

In general, the landslide hazard in the case of the two studied landslides is not very high, since we recorded no increase in landslide activity from year to year, which was typical for the Polish landslide cases. The exception is 2023, when a significant increase in the landslide activity was recorded at landslide 1 (Figure 4B). In the future, further precipitation may cause this landslide to become increasingly active. However, other studies conducted using the proposed method [29] do not predict a sudden activation of the studied landslides.

The landslide hazard method presented in this paper, based on the method described by Malik et al. [29], is helpful for landslides that occur independently of climatic conditions and geological structure. The trees exhibit small ground movements that have increased over the last few years (as documented with our method for the last 10 years). In that case, this indicates that the slope is becoming increasingly prone to the occurrence of a catastrophic landslide. Of course, some landslides are triggered suddenly without any minor preparatory movements. This is particularly true for massive landslide disasters, such as those caused by high-magnitude earthquakes, when thousands of landslides are formed in an instant under the influence of a powerful environmental impulse [54,55].

Notwithstanding this limitation, which applies to all other methods of landslide hazard assessment, the method used in this paper can also be used in the case of landslide hazard analysis on every forested slope. Additionally, in the case of a forested slope, it is practically impossible to use methods such as radar interferometry; therefore, the proposed method can be complementary to methods using satellite systems. To make the method more precise, more tree ring patterns formed by trees before landslide catastrophes in different parts of the world should be collected.

The results of the conducted study complement the work aimed at determining landslide susceptibility in the Vernazza catchment, which was calculated based on the occurrence of shallow landslides in 2011 [46]. The authors did not include in their determination of susceptibility the possible triggering of deep-seated landslides. These landslides may be triggered under very different conditions from those experienced in 2011, for example, under the influence of long-lasting precipitation. However, such meteorological conditions are relatively rare in Liguria.

One of the most significant advantages of the dendrochronological method is the possibility of obtaining long data series—several decades long, and, in favourable conditions, even several centuries long. Currently, only the dendrochronological method enables the collection of such long data series on landslide activity. Additionally, when a landslide is covered with trees, it is possible to collect multiple samples from the landslide and determine its spatial variability, which allows for the development of landslide activity maps [56]. The use of the dendrochronological method for dating landslides also has limitations. The most significant issue, of course, is the lack of trees on the landslide slope, which renders the method impossible to use. Dendrochronological dating is often delayed by one year in relation to the movement caused by the landslide. It occurs when a landslide episode happens in winter, after the growing season, e.g., in December, and the trees record the landslide movement in the following year. Often, the reaction of trees is delayed by a strong response to landslides, occurring one or two years after the landslide episode. This happens when trees are damaged during a landslide, such as when their roots are torn out. In such cases, trees produce reduced rings or sometimes a phenomenon of missing rings occurs. In such cases, the dendrochronological record of the landslide is delayed in relation to the occurrence of the landslide episode. Another limitation is the low resolution of the method, which is 1 year, whereas radar interferometry, for example, has a much higher resolution of several to a dozen days.

## 5. Conclusions

1 Studied landslide activity is recorded as minor landslide episodes that change over time and are probably controlled by rainfall events. Years with high landslide activity (very high landslide activity in 2015, 2020, and 2023, and high landslide activity in 1993, 1997, and 2006) usually correspond to rainfall events or years.
2 Within the two studied landslides located in the same catchment, increased landslide activity occurred at similar times. However, the dominant episodes occurred in different years; for example, in 2020, 50% of the trees in one landslide reacted to the landslide, while only 20% of the trees reacted in the other. This means that information on landslide activity and landslide hazard should be generalised with caution, even within a single catchment area.
3 Trees growing on landslides did not record high landslide activity immediately after the catastrophic event in 2011. An increase in landslide activity occurred three years later, in 2014. Since that year, landslide activity has been significantly higher than during the period preceding the 2011 catastrophic event. Perhaps the trees did not register a landslide due to partial destruction of their root systems or a change in local water relations after the 2011 disaster, resulting in reduced and missing rings. This suggests that extreme events may occur in tree ring series with a specific lag time. The high landslide activity recorded by trees in the period 2014−2023 may be due to the significant erosion of material from the catchment in 2011, accompanied by a significant transformation of the catchment’s relief. These changes may have resulted in a loss of stability of deep-seated landslides.
4 There is no high risk of sudden triggering of the studied landslides. The landslide activity recorded in trees does not show an increase, unlike what was reported for the Polish landslides, where the trees registered an increase in activity from year to year in the years preceding the landslide disaster in 2010. The exception is 2023, when a significant increase in activity was recorded at one of the studied landslides. However, this increase occurred only in a single year, and there is no sustained trend of increased preparatory landslide movements, unlike the case of Polish landslides.

## Data Availability

The original contributions presented in this study are included in the article. Further inquiries can be directed to the corresponding author.
